# Spatial mapping of humeral head bone density

**DOI:** 10.1016/j.jse.2017.03.006

**Published:** 2017-09

**Authors:** Hamidreza Alidousti, Joshua W. Giles, Roger J.H. Emery, Jonathan Jeffers

**Affiliations:** a Department of Mechanical Engineering, Imperial College London, London, UK; b Department of Surgery & Cancer, Imperial College London, London, UK

**Keywords:** Shoulder arthroplasty, humeral bone density, short-stem devices, implant fixation, humeral component, implant design, implant loosening

## Abstract

**Background:**

Short-stem humeral replacements achieve fixation by anchoring to the metaphyseal trabecular bone. Fixing the implant in high-density bone can provide strong fixation and reduce the risk of loosening. However, there is a lack of data mapping the bone density distribution in the proximal humerus. The aim of the study was to investigate the bone density in proximal humerus.

**Methods:**

Eight computed tomography scans of healthy cadaveric humeri were used to map bone density distribution in the humeral head. The proximal humeral head was divided into 12 slices parallel to the humeral anatomic neck. Each slice was then divided into 4 concentric circles. The slices below the anatomic neck, where short-stem implants have their fixation features, were further divided into radial sectors. The average bone density for each of these regions was calculated, and regions of interest were compared using a repeated-measures analysis of variance with significance set at *P* < .05.

**Results:**

Average apparent bone density was found to decrease from proximal to distal regions, with the majority of higher bone density proximal to the anatomic neck of the humerus (*P* < .05). Below the anatomic neck, bone density increases from central to peripheral regions, where cortical bone eventually occupies the space (*P* < .05). In distal slices below the anatomic neck, a higher bone density distribution in the medial calcar region was also observed.

**Conclusion:**

This study indicates that it is advantageous with respect to implant fixation to preserve some bone above the anatomic neck and epiphyseal plate and to use the denser bone at the periphery.

Short-stem humeral component designs have been introduced by several manufacturers in the past few years.^6^, ^8^ The benefits of this type of design (Fig. 1) include decreased bone resection compared with conventional stemmed implants and the ability to replicate the native humeral head center without compensating for a patient's variable humeral shaft offset.^2^ A drawback of such designs is that they rely on a smaller proximal region for fixation with a less advantageous lever arm, which is not located as far down the shaft of the humerus, compared with traditional stemmed designs. Currently available short-stem designs resect the humeral head and achieve fixation in bone distal to the resection plane in the trabecular metaphyseal region. The density of the bone in this region is therefore important for achieving adequate component fixation. In fact, Favre et al,^3^ using cadaveric humeri, showed that in a short-stem device, micromotion between bone and implant may increase significantly with decreased apparent bone density. They showed that when bone density is lower than 0.1 g∙cm^−3^, an implant may experience micromotions above the 150-µm threshold accepted to result in bone growth.^7^, ^12^

**Figure 1:**
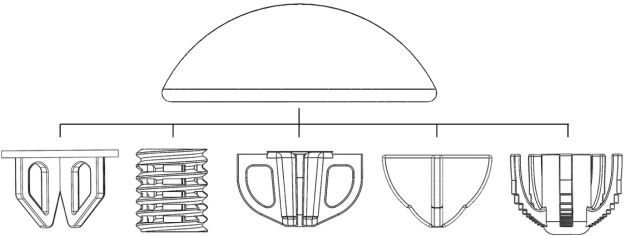
Diagram of the most common short-stem humeral components. The hemispherical head is assembled to a variety of stem designs shown in the figure using a taper fit mechanism. The stem is press fitted into the cancellous bone beneath the anatomic neck cut.

A number of studies have investigated the bone density distribution in the proximal humerus. A summary of their methodologies and findings is shown in Table I. In a study on dissected proximal humeri using bone mineral densitometry and an indentation test, Saitoh et al^14^ showed that the proximal part of the humeral head exhibited the greatest amount of bone mineral density and the humeral neck had approximately half the bone mineral density of the humeral head. In addition, they also showed that the cancellous bone of the neck had only one-third the mechanical strength of the humeral head in the indentation test. In a volumetric bone mineral density assessment of 20 cadaveric bones, Tingart et al^16^ showed that trabecular bone has significantly higher density in the proximal posterior portion of the articular surface. Yamada et al^21^ performed a computed tomography (CT) study of 40 patients and found that bone density was higher on the medial side of the humeral head, especially near the articular region. Hepp et al^5^ investigated bone strength rather than density by slicing 24 cadaveric humeri and measuring bone strength by indentation. They showed that medial and posterior aspects of the proximal humerus had the highest bone strength. In addition, they found that the greater and lesser tuberosities and the central area of the proximal head had the lowest bone strength. Barvencik et al^1^ studied age-related changes in bone density in 60 cadaveric proximal humeri. They investigated bone density using x-rays and found that the most superior and medially located part of the humerus had highest bone density independent from age. They also found that the most prominent decrease in bone density due to age was observed in the region of the greater tuberosity.

**Table I:** Schematics of studies and their methodologies for carrying out bone density and strength measurement for the proximal humeral head

Study	Method	Findings
Barvencik et al^1^ • Bone mineral measurement using histomorphometric analysis and x-rays in A to L regions on 1 centered coronal humeral head slice	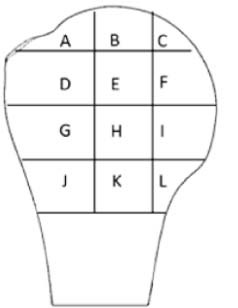	The most superior and medially located part of the humerus (C, B, and F) had highest bone density.
Hep et al^5^ • Bone mineral measurement using histomorphometric analyses for 1, 3, 5, and 7 slices • Indentation test on 2, 4, 6, and 8 slices in A to E regions	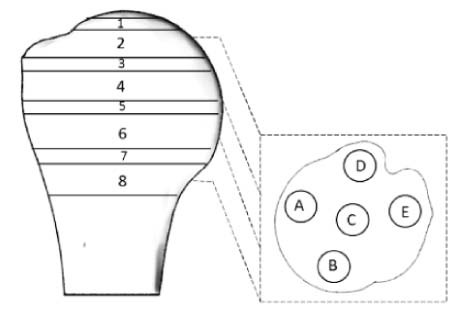	Medial (A) and posterior (B) aspects of the proximal humerus had the highest bone strength. The greater (E) and lesser (D) tuberosities and the central (C) area of the proximal head had the lowest bone strength.
Saitoh et al^14^ • Bone mineral densitometry on the entire region of each of 3 slices • Indentation test on 3 slices in A to I regions	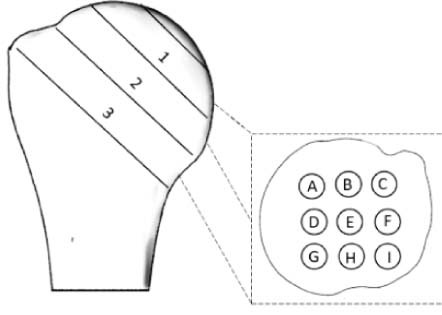	Bone above anatomic neck (1) showed twice bone mineral and 3 times higher mechanical strength than bone of the humeral neck (3). Posteroinferior regions were mechanically stronger than other regions.
Tingart et al^16^ • Bone mineral densitometry on the entire region of each of 6 slices • Regional bone mineral densitometry for 1 head middle slice in regions A to G	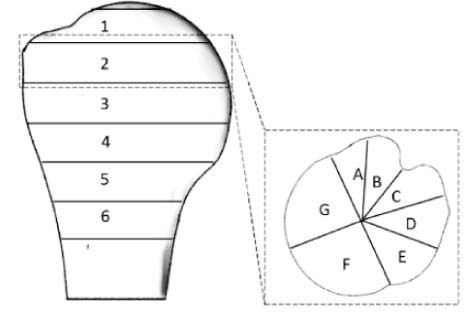	Bone has significantly higher density in the proximal (1, 2, and 3) posterior (D and E) portion of the articular surface.
Yamada et al^21^ • Bone density measurement based on Hounsfield unit values on each computed tomography slice in the region shown • Regional density measurement in each slice for A and B regions	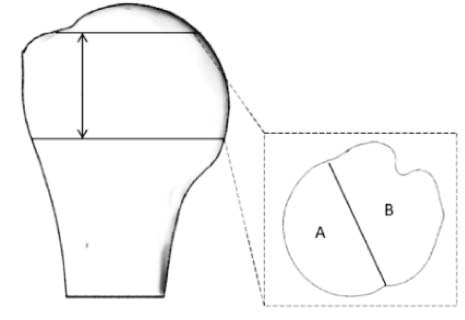	Bone density was higher on the medial side (A) of the humeral head, especially near the articular region.

These studies provide valuable information on the spatial distribution of bone in the proximal humerus for screw fixation, rotator cuff repair suture anchors, or conventional stemmed humeral devices. However, they report data in the transverse plane (more appropriate for conventional stemmed humeral devices) or with limited data resolution in this volume of interest. Hepp et al^5^ reported strength from 5 points in 4 transverse slices in the proximal humerus, Yamada et al^21^ divided the CT data into 2 areas (medial and lateral) for transverse slices of the proximal humerus, and Barvencik et al^1^ assessed a single coronal slice of the proximal humerus. Tingart et al^16^ did report their data relative to the humeral neck, but only in 1 slice that was perpendicular to the long axis of the shaft. A summary of the measurement location of these studies is shown in Table I. As a result, the data provided by these studies are of limited use in relation to short, proximally fixed humeral designs that are orientated in the plane of the head-neck junction, with fixation features protruding around 20 to 40 mm perpendicular to that plane. For such devices, the spatial density map therefore needs to be reported in a reference frame relative to the anatomic neck and to provide high-resolution density mapping in the volume of bone proximal to this plane and 20 to 40 mm distal to this plane.

A spatial map of humeral bone density in the volume of bone where devices with proximal fixation achieve fixation would therefore be useful to surgeons by providing a guide for the positioning of anchoring features of existing implants and could prove to be a critical resource for implant designers seeking to improve the fixation features of future humeral components by using denser regions of the bone. Therefore, this study aimed to provide a detailed map of bone density in the proximal humerus, specifically in the bone distal to the humeral neck where these devices achieve fixation. The null hypothesis is that there is no statistically significant relationship between the bone density and the spatial location in proximal to distal, central to peripheral, and radial directions in the humeral head.

## Method

Eight CT scans of independent cadaveric humeri specimens with mean ± standard deviation age of 71 ± 10 years (range, 59-83 years; 4 male) were used. There was no evidence of degenerative joint disease or osteoporosis in the specimens. The CT scans were carried out using a Toshiba (Tokyo, Japan) Aquilion 32 machine. Standard phantoms of Delrin, nylon, and polypropylene provided by the manufacturer were used to calibrate the machine for bone, soft tissue, and fat according to the manufacturer's protocol. Scans had a resolution of approximately 0.5 × 0.5 × 0.9 mm and were manually segmented in the Mimics software package (version 15.0; Materialise NV, Leuven, Belgium) to generate solid models. The solid models were then discretized into 1-mm tetrahedral elements in the 3-matic software package (version 7.0; Materialise NV), and material properties were assigned on the basis of the local Hounsfield unit from the CT scan. A rigid polyurethane foam phantom with a known density of 0.29 g∙cm^−3^ provided a reference to calibrate Hounsfield values and ensured consistency between CT scans. The apparent bone density was calculated using the recorded Hounsfield unit (HU) values and the relationship described by Rho et al^13^ in which CT value and the apparent bone density in the proximal humerus were found to follow the formula

$$\rho \left(g\cdot cm^{-3}\right)=0.131+\left(0.000624*HU\right)$$

where apparent bone density is defined by wet weight divided by volume of the overall physical dimension of a given specimen. For each CT scan, the lower limit that distinguished the fatty marrow from bone tissue was established. Those cloud points that fell below this limit were excluded from the analysis.

**Figure 2:**
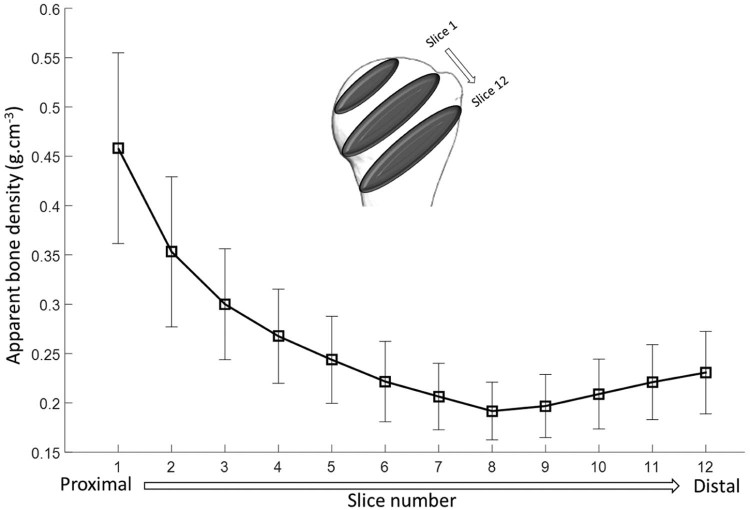
Variation of bone density from the proximal to distal region across slices parallel to the anatomic neck. The range and the orientation of the slices are shown, with the anatomic neck at slice 6.

**Figure 3:**
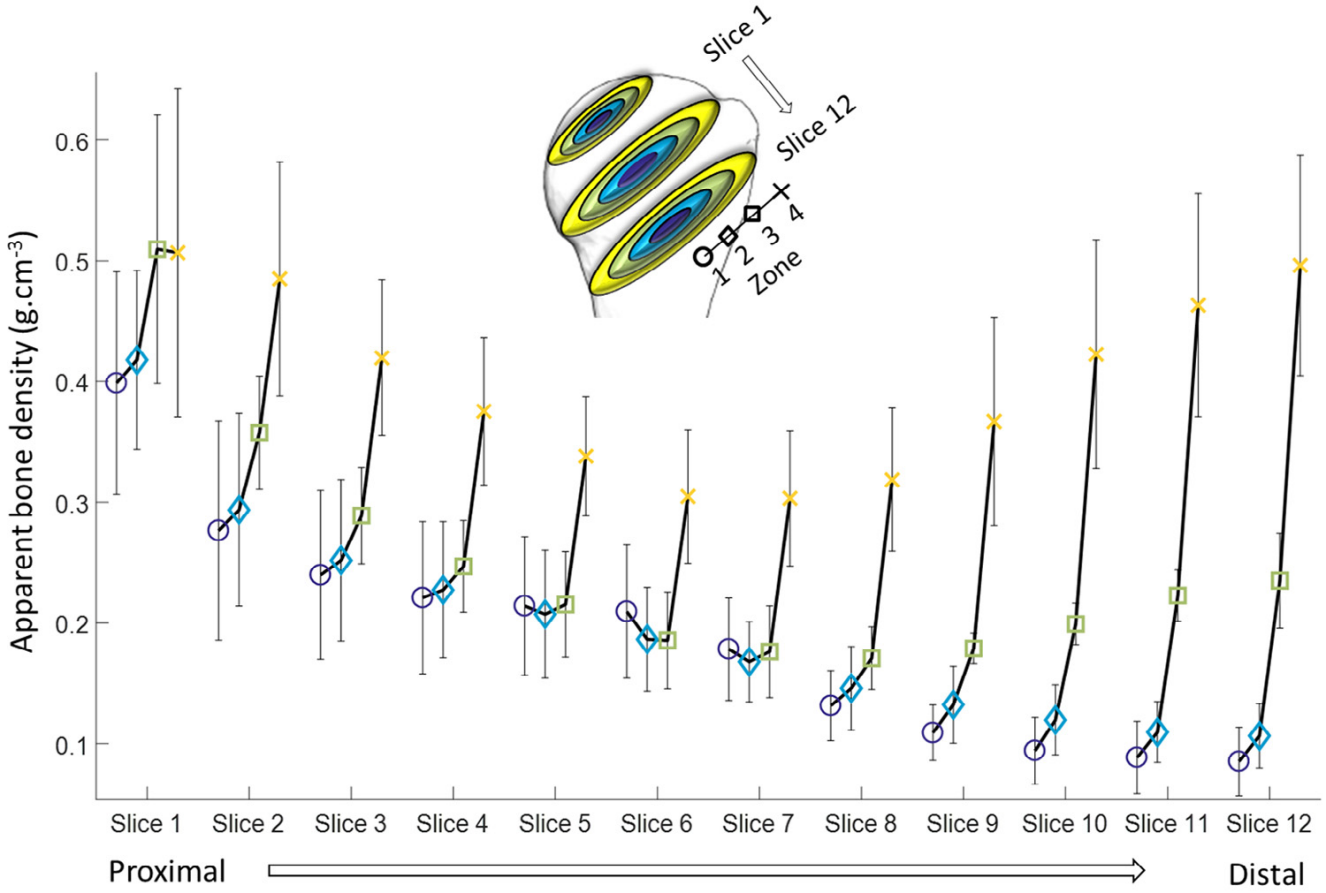
Variation of bone density from central to peripheral zones for each slice parallel to the anatomic neck. The range and the orientation of the slices are shown, with the anatomic neck at slice 6.

In MATLAB (R2015a; MathWorks Inc., Natick, MA, USA), each element centroid was calculated and the corresponding apparent bone density in that element was assigned to its centroid. This provided a cloud of points in space with density values. The data were then rearranged and grouped by dividing the humeral head into 12 slices parallel to the humeral neck starting from the most proximal region to the distal regions beneath the epiphyseal plate (Fig. 2). The humeral neck was identified by an experienced surgeon on the basis of anatomic landmarks. Slices were made such that the sixth slice coincided with the anatomic neck. Each slice was then divided into 4 concentric zones (Fig. 3). Using the distal to proximal and concentric subdivisions, the overall spatial variation and specific interactions between geometric variables were investigated using descriptive and inferential statistics (see Statistics section).

**Figure 4:**
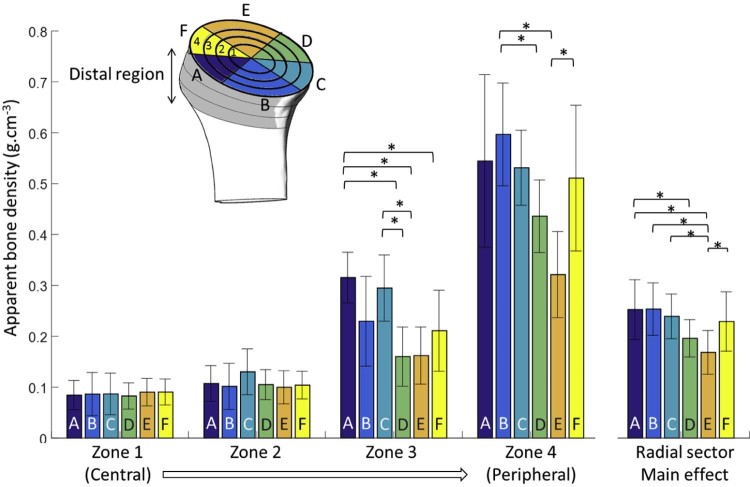
Graph of mean bone density stratified by concentric zone (1-4) and radial sectors (A-F). *Asterisks* and *brackets* represent significant comparisons (*P* < .05) between radial sectors for a given concentric zone. Also, note that data are for the average of the 6 slices distal to the anatomic neck.

The majority of current short-stem implants place their fixation entities in the bone below the humeral anatomic neck without recommending an optimal orientation. Therefore, to investigate whether there is a meaningful difference in the apparent bone density at different orientations within each slice, the concentric zones in this distal region were further divided into 6 radial sectors (Fig. 4) and assessed using descriptive and inferential statistics. For each of these subregions, apparent bone density values were calculated by averaging the density values of the cloud of points located within their volume. In deciding on the number of subvolumes—and thus their size—it was ensured that the average value calculated from the cloud of points for each subvolume did not mask important local variations in properties. This was achieved by defining the number of subvolumes using the criterion that the standard deviation of the values of all points within a subvolume must not exceed 10% of the average.

### Statistics

To provide a comprehensive understanding of the spatial mapping of the calculated apparent bone density values, they were first assessed using descriptive statistics for all of the described subdivisions (ie, 12 slices with 4 concentric zones on each slice). Subsequently, statistical differences in observed spatial variations were analyzed using 2 two-way repeated-measures analyses of variance (RM-ANOVA) in SPSS 22 (IBM, Armonk, NY, USA) with factors of proximal to distal slices (3 levels) and concentric zones (4 levels), concentric zones (4 levels), and radial sectors (6 levels). For proximal to distal slices, the 12 proximal to distal slices were grouped and averaged as blocks of 4 (hereafter termed proximal, middle, and distal) as these were typically 1 mm thick and the analysis of statistical difference for regions of this small size was not considered clinically meaningful. For concentric zones, only the region below the anatomic neck was considered as this is where current short-stem fixation occurs, and the values of these 6 slices were averaged together as implant fixation features pass through all of these. For each RM-ANOVA, significance was set at *P* < .05 as well; follow-up post hoc tests with a Bonferroni correction and analyses of interactions were performed when appropriate. Power analysis indicated that a sample size of 8 humeri was required to achieve 80% power for each of the RM-ANOVA statistical analyses. For this power analysis, we chose our clinically meaningful difference in bone density to be 15%, which falls between the 10% and 20% values previously reported in the literature,^9^, ^15^ and our group standard deviations were ±0.037 g∙cm^−3^ as taken from pilot specimens.

## Results

Considering each proximal to distal slice as a whole, it was found that apparent bone density was at its maximum at slice 1 (0.45 g∙cm^−3^), was at its minimum at slice 8 (0.20 g∙cm^−3^), just below the anatomic neck, and increased to 0.24 g∙cm^−3^ by the most distal slice (Fig. 2).

When slices were subdivided into 4 concentric zones (Fig. 3), it was observed that by moving from proximal to distal, changes in bone density differed in the central and peripheral regions. The peripheral zone (zone 4), which contains the cortex, had the highest density value in all slices, but a trend for increasing density from zone 1 to 3 was also observed (ie, the density increased progressively from the central to the peripheral region of the bone). This effect is a result of the continual decrease in bone density in the central zones from proximal to distal in contrast to that of the peripheral zones, where, after decreasing, density starts increasing in regions below the anatomic neck, from slice 8 to 12 (Fig. 3).

### Statistical comparisons

**Figure 5:**
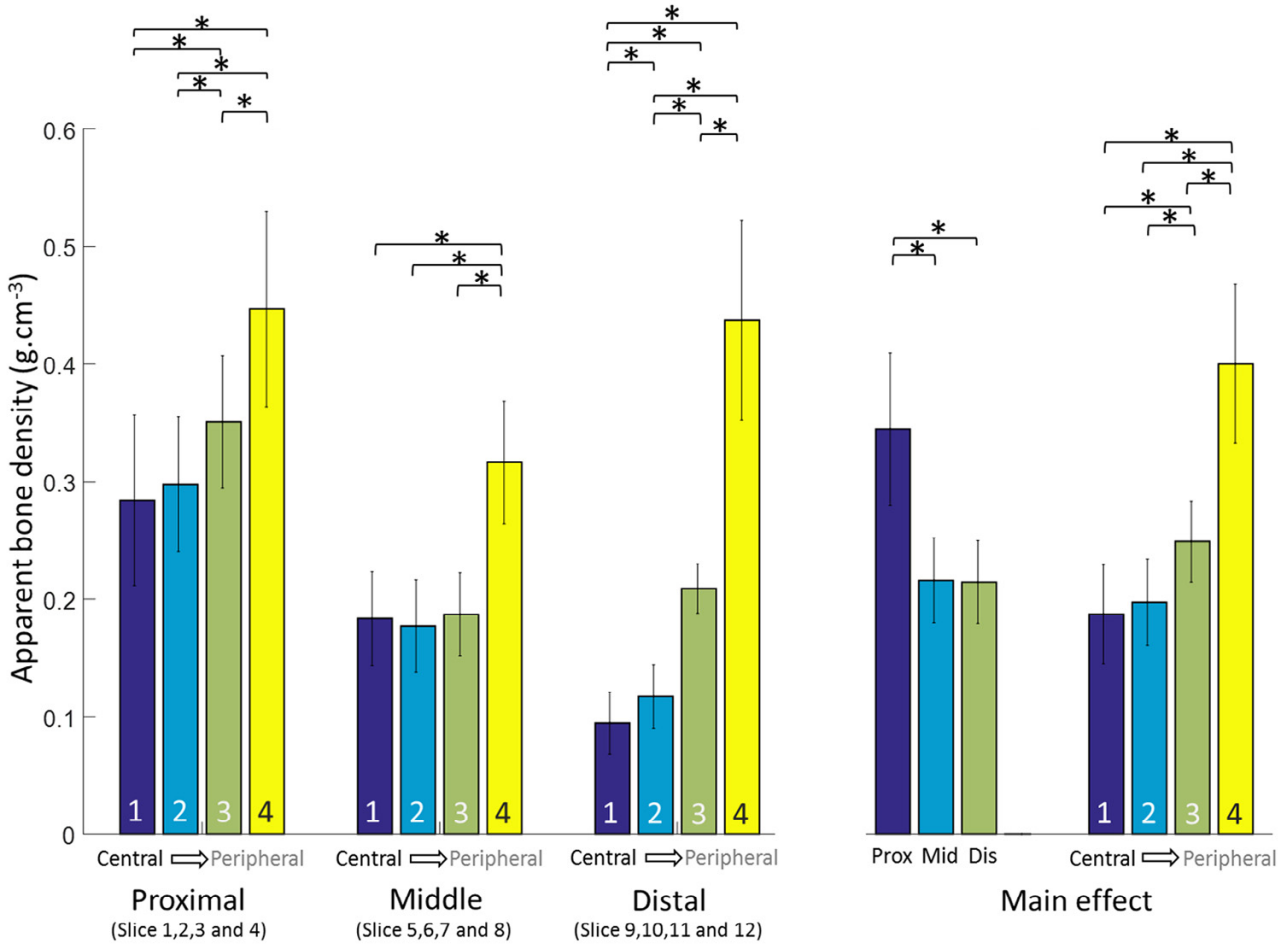
Graph of mean bone density stratified by proximal to distal (proximal, middle, distal) position and concentric zone (1-4). *Asterisks* and *brackets* represent significant comparisons (*P* < .05) between concentric zones for a given proximal to distal region. Also, note that slice data were grouped and averaged into distal, middle, and proximal regions to make comparisons more clinically meaningful as described in the Statistics section.

Statistical analysis of these data using 2-way RM-ANOVA rejected the null hypothesis and demonstrated a number of important regional trends and statistically significant differences. Moving from proximal to distal in the humeral head was found to produce a significant main effect on bone density (both *P* < .001) (Fig. 5). Specifically, when averaged across all concentric zones, the proximal region was significantly more dense (0.34 ± 0.06 g∙cm^−3^) than the middle (0.22 ± 0.04 g∙cm^−3^; *P* < .001) and distal (0.21 ± 0.03 g∙cm^−3^; *P* < .001) regions. Similarly, when averaged across all proximal to distal regions, all concentric zones were significantly different from one another (differences: 0.06-0.22 ± 0.02-0.04; .001 < *P* ≤ .001) except zone 1 to 2 (difference: 0.01 ± 0.01; *P* = .059). However, it was also found that proximal to distal and central to peripheral positioning significantly interact (*P* < .001), such that bone density is significantly reduced in concentric zones 1 and 2 from approximately 0.28 g∙cm^−3^ to 0.1 g∙cm^−3^ by moving from proximal to distal; but for zones 3 and 4, there is a characteristic decrease and increase from proximal to middle and middle to distal, respectively. However, in the middle region of the humerus, where the anatomic neck is located, apparent bone density in zone 3 drops below 0.2 g∙cm^−3^, and in the distal region it has a value of just above 0.2 g∙cm^−3^. More detailed analysis of this interaction found that there were numerous significant differences between the various levels of each of the 2 factors, with proximal and distal regions producing similar patterns (if not magnitudes) of differences and only zone 4 being different from the others in the middle region.

As mentioned previously, to further investigate bone density variations in distal regions, where implant fixation entities are normally placed, 6 slices below the anatomic neck were averaged together and then split into 4 concentric zones and further subdivided into 6 radial sectors (Fig. 4). This 2-way RM-ANOVA rejected the null hypothesis and found that both the concentric zone (*P* < .001) and radial sector (*P* < .001) of a bone region had a significant main effect on bone density. With respect to the main effect of the radial sector, it was found that bone density increases from its minimum at the most lateral sector in the vicinity of the greater and lesser tuberosities (sector E: 0.17 ± 0.04 g.cm^−3^) as you rotate in either direction until it reaches its maximum in the medial calcar (sector B: 0.25 ± 0.05 g∙cm^−3^). This pattern resulted in significant differences between sector E and all others (differences: 0.06-0.09 ± 0.02-0.04; .003 ≤ *P* ≤ .049) except sector D (difference: 0.02 ± 0.3; *P* = 1.000), which is adjacent. In addition, sector A was significantly more dense than sector D (difference: 0.06 ± 0.03; *P* = .026). Furthermore, there was a significant interaction between the 2 factors (*P* = .004) such that there were no statistically significant differences between radial sectors in central zones; however, in peripheral regions (zone 3 and 4), there were significant differences, and each zone demonstrated the characteristic pattern described for the main effect before (ie, lower density laterally and greater medially).

## Discussion

This study showed that apparent bone density is higher proximal to the anatomic neck of the humerus. Apparent bone density also increases from central to peripheral regions. This difference in bone density from central to peripheral zones is more pronounced in regions below the anatomic neck. These regions of bone are therefore most suitable to achieve fixation of proximally fixed humeral implant designs. Furthermore, below the anatomic neck, bone density has the greatest density in the medial calcar region (ie, the 3- to 9-o'clock positions on the clock face) and the lowest at the lateral humeral head adjacent to the greater and lesser tuberosities (ie, the 11- to 1-o'clock positions). Proximally fixed humeral components usually have a cruciform fixation keel that can be positioned to avoid this low-density region.

The current findings are in agreement with the finding of Saitoh et al,^14^ who showed that the mineral bone density in humeral neck was approximately half the bone mineral density of the humeral head (Fig. 5). This study is also comparable to that of Barvencik et al,^1^ who showed higher density in proximal regions when a slice of the proximal humerus was viewed in the coronal plane. Yamada et al^21^ also found high local bone density on the medial side of the humerus and showed that regions in the vicinity of the lesser and greater tuberosities contained less bone tissue. Similarly, Hepp et al^5^ showed higher densities in the medial and posterior regions of the proximal humerus. The increase in apparent bone density from central to periphery regions demonstrated in this study is also in agreement with earlier findings that the strength and rigidity of cancellous bone significantly increase within 2 to 5 mm of the cortical wall.^10^ Our data indicate that this is more evident in distal regions, where a sharp increase in density is observed from zone 3 to 4.

Short-stem implants are designed to give surgeons greater access to the glenoid compared with resurfacing devices while preserving more bone than traditional stemmed implants; however, the disadvantage of these implants is that they must achieve fixation over a smaller surface area and with a less advantageous lever arm. Current generation short-stem designs require the entire humeral head to be resected at the anatomic neck. Our data indicate that this may sacrifice regions of higher quality bone proximal to the anatomic neck that could be used to achieve better fixation and thereby allow lower profile fixation features, for example, a midhead resection that allows access to the glenoid while retaining some bone proximal to the anatomic neck. In addition, the majority of short-stem designs consist of a primary central fixation keel below the anatomic neck, with fins or webs extending from this central feature. The current study shows that below the anatomic neck, the central portion of the humerus may have a density just around 0.1 g∙cm^−3^, which has been shown to be an indication for increased micromotion above the commonly accepted value of 150 µm.^3^ This micromotion threshold may be exceeded in the shoulder for short-stem devices, before osseointegration for normal activity. Telemeterized implant data indicate that the joint force can reach 850 N (120% body weight of 70-kg patient) by placing a 2-kg object on a shelf,^18^ which has been shown to create micromotions up to 270 µm for a short-stem device.^3^ Our data indicate that the peripheral bone has greater density, and this also may be used for lower profile peripheral fixation features of the humeral component. Our data also show that the highest density bone is located in the outer third and fourth concentric rings of every slice, particularly those located below the anatomic neck. This observation may suggest that any peripheral fixation should have its features located at around 75% of the radial distance extending from the implant center to its rim. Because the bone slices are always divided into 4 concentric regions regardless of the patient's size and shape variability, the denser bone will not be missed in some individuals for such a peripheral design. Locating fixation features in the densest bone may extend the indication for a humeral replacement procedure in patients with decreased bone density. In fact, Hall and Rosser^4^ showed that loss of bone substance due to osteoporosis occurs centrally beneath the epiphyseal plate and in the greater tuberosity, and the peripheral regions remain less affected.

Wirth et al,^19^ using finite element simulations developed from micro-CT scans of the humeral trabecular bone, have demonstrated that an implant-bone construct located in the central region of the humerus trabecular bone has less structural stiffness than those placed peripherally. In addition, Favre et al,^3^ using cadaveric humeri and displacement-measuring transducers, have shown that micromotion in short-stem designs significantly increases as the trabecular apparent bone density decreases. The data in the current study may therefore be useful for implant design but are also useful for positioning of existing designs. It may be advantageous to place at least 1 of the peripheral fixation entities in the stronger bone in the medial and posterior regions. This may be especially advantageous for patients who experience a marked decrease in bone density in the region of the greater tuberosity because of age while their bone density remains unchanged in the medial and posterior regions independent of age and sex as described by Barvencik et al.^1^ In addition, Shah et al,^15^ using osteoblasts derived from age-matched and paired humeral head samples with osteoarthritis and osteoporosis, showed that cortical and subchondral bone had greater proangiogenic (higher levels of vascular endothelial growth factor A messenger RNA and protein release) capacity and fracture healing characteristics compared with trabecular bone. Their trabecular bone sample taken from the central regions distal to the anatomic neck also consistently showed slower osteoblast proliferation. These findings suggest that denser proximal bone (close to subchondral bone) and peripheral bone (close to cortical bone) identified in the current work may also benefit from this greater biologic activity and therefore have greater osseointegration potential compared with the central and distal cancellous bone.

This study has a number of limitations. First, the study includes a small sample size of 8 humeri. However, our power analysis indicated that this was sufficient to identify clinically significant differences of 15% in bone density. Second, the specimens were exclusively healthy joints. However, the proximally fixed devices discussed in the study are aimed at an earlier intervention than conventional total shoulder arthroplasty; thus, when these devices are used, the bone will not have reached the deterioration associated with end-stage disease. A future follow-up study will use the same methodology to investigate density distribution preoperatively in actual patients. Third, apparent bone density was not directly measured through in vitro experiments but instead using CT data; however, Rho et al^13^ found a strong correlation between CT-based apparent density and physically measured apparent density of the proximal humerus. It has also been shown that bone density is a good predictor of implant performance^11^, ^17^, ^19^, ^20^ and can predict bone Young modulus with a good correlation.^13^ This was confirmed by the regions of high bone density found in this study that correspond to the regions with higher mechanical strength shown by Hepp et al.^5^ Fourth, our study did not distinguish between the cortical and trabecular bone in the higher density peripheral region (concentric zone 4). However, the trend of bone density increase from central to periphery clearly demonstrates higher bone density in the trabecular bone in the vicinity of the cortical bone as suggested by Pilliar et al.^10^ In addition, the thickness of the cortical shell in proximal humerus was measured to vary between 1 and 2 mm in all specimens, and the highest thickness measured is still only approximately one-fifth the thickness of the outer concentric zone (zone 4). This indicates that a large portion of the concentric zone 4 is occupied by trabecular bone, and therefore the higher density in this region is not wholly attributable to the cortical shell. Furthermore, the increase in bone density in the peripheral regions is strongly evident in zone 3, where there is no cortical bone present.

## Conclusions

This study is the first to comprehensively map humeral bone density and has shown that there are significant regional differences, with the most pronounced effects being stronger bone proximally and peripherally as well as in the medial calcar region. Therefore, any new humeral implants should use these stronger regions of bone located above the anatomic neck, which have also been shown to exhibit biologically better osseointegration properties,^15^ and at the periphery by incorporating strategically located fixation features.

## Disclaimer

This work was funded by the Translation Award received from Wellcome Trust.

Hamidreza Alidousti, Roger J.H. Emery, and Jonathan Jeffers may receive benefits from patent 1512277.3 (pending) related to this work. Joshua W. Giles, his immediate family, and any research foundations with which they are affiliated have not received any financial payments or other benefits from any commercial entity related to the subject of this article.
